# Beyond Alkaloids: Novel Bioactive Natural Products From *Lobelia* Species

**DOI:** 10.3389/fphar.2021.638210

**Published:** 2021-03-08

**Authors:** Qinfang Zheng, Ye Wang, Shuihan Zhang

**Affiliations:** ^1^ Hunan Academy of Chinese Medicine, Hunan University of Chinese Medicine, Changsha, China; ^2^ Key Laboratory of Dong Medical Research of Hunan Province, Hunan University of Medicine, Huaihua, China; ^3^ 2011 Collaboration and Innovation Center for Digital Chinese Medicine in Hunan, Changsha, China

**Keywords:** lobelia, alkaloids, natural products, lobeline, bioactive compounds

## Abstract

In this work, we reviewed the progress in the phytochemical and biological investigations of bioactive components derived from medicinally valuable *Lobelia* species. In the last 60 years, *Lobelia* has garnered significant attention from the phytochemist from around the world, majorly due to the discovery of bioactive piperidine alkaloids (e.g., lobinaline and lobeline) in the early 1950s. Later, lobeline underwent clinical trials for several indications including the treatment of attention deficit hyperactivity disorder and a multicenter phase three trial for smoking cessation. Subsequently, several other alkaloids derived from different species of *Lobelia* were also investigated for their pharmacological characteristics. However, in the last few years, the research focus has started shifting to the characterization of the other novel chemical classes. The major shift has been noticed due to the structurally similar alkaloid components, which essentially share similar pharmacological, physicochemical, and toxicological profiles. In this review, we present an up-to-date overview of their progress with special attention to understanding the molecular mechanisms of the novel bioactive components.

## Introduction

*Lobelia*, named after the botanist Matthias de lobel, is a large genus of medicinally valuable flowering plants belonging to the Campanulaceae family comprising more than 450 species distributed predominantly in tropical and temperate regions of the world. Several species of *Lobelia* are traditionally used in different folk medicine to treat various diseases. Among them, *Lobelia inflata*, *Lobelia nicotianaefolia*, *Lobelia cardinalis, Lobelia chinensis*, *Lobelia laxiflora*, *Lobelia trigona* Roxb., *Lobelia siphilitica* L., *Lobelia sessilifoilia* Lamb, *Lobelia polyphylla*, and *Lobelia pyramidalis* Wall. are some of the frequently used *Lobelia* species. The phytochemical researches of these species led to the discovery of several novel bioactive secondary metabolites.

**FIGURE 1:**
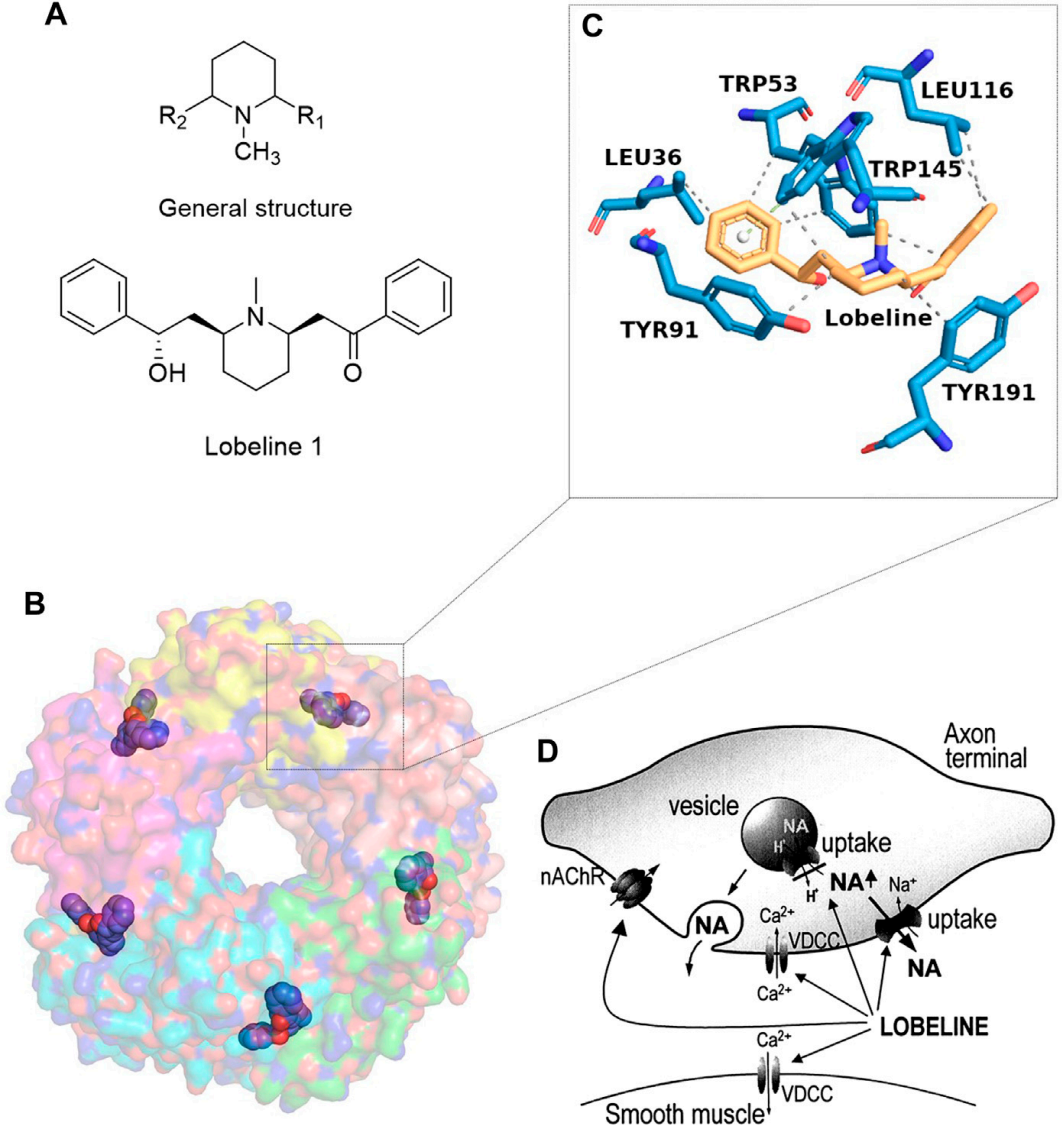
Structure and molecular mechanism of lobeline. **(A)** Chemical structure. **(B, C)** Molecular interaction of lobeline bound to the human α7-nAChR (Spurny et al., 2015). **(D)** A schematic diagram of the multiple cellular mechanisms of lobeline on the release of norepinephrine taken from the reference (Sántha et al., 2000).

The earliest scientific report on the pharmacological use of *Lobelia* species in diseased conditions dates back to 1828 (Andrew, 1828). More than a century later (in 1950), Tondeur and Charlier reported the extraction and pharmacological activities of alkaloids from *Lobelia suavibracteata* (Haumann) and *Lobelia giberroa* (Hemsl.) (Charlier and Tondeur, 1950; Tondeur and Charlier, 1950). In the 1950s, other studies began to reveal the distinctive pharmacological properties of the alkaloid constituents derived from different *Lobelia* species (Lendle and Richter, 1950). One of the alkaloids that garnered major attention was lobeline 1 (Figure 1), which was primarily derived from the aerial parts of *Lobelia inflata,* (Kaczmarek and Steinegger, 1959; Saldanha et al., 1959). In the subsequent decades, the studies on the prospective evaluation of the use of lobeline have seen unprecedented advances, including but not limited to smoking cessation (Glover et al., 2010; Stead and Hughes, 2012), treatment of drug and alcohol abuse (Bell et al., 2009; Hart et al., 2010; Roni and Rahman, 2017), adult ADHD (Martin et al., 2018), anti-depressant (Roni and Rahman, 2015), anti-epileptic (Tamboli et al., 2012), and Parkinson’s disease (Li et al., 2014).

For example, in their study, Roni and Rahman noticed that adult C57BL/6J mice when treated with once daily dose of lobeline (1 mg/kg, s.c.) in the last 14 days of their exposure to chronic unpredictable stress (CUS) for 6 weeks produced antidepressant-like effects. They proposed targeting brain nicotinic acetylcholine receptors (nAChRs), brain-derived neurotrophic factor (BDNF) expression and/or cell proliferation in the hippocampus to be the most likely cause of the observed antidepressant-like effects of lobeline (Roni and Rahman, 2015). Saline was used as the negative control. Hart et al. reported that at doses of 1.0 mg/kg and 3.0 mg/kg subcutaneous injections of lobeline attenuates self-administration of intravenous (i.v.) heroin (3,6 diacetylmorphine HCl) infusions (18 μg/kg) in male Sprague-Dawley rats suggesting their use in pharmacotherapy for opioid abuse (Hart et al., 2010). In both studies, however, they did not make a comparison of the effect of lobeline with any reference standard.

Martin et al. reported the results of a preclinical study in which they studied the effect of lobeline in adult ADHD (Martin et al., 2018). In their study, they subjected a total of nine adults (5 females, 4 males, ages 23–41 [31.11 ± 7.08 years]) to a 7-day protocol of oral administration of methylphenidate capsule (0, 15, or 30 mg, p.o.) followed by sublingual (s.l.) administration of lobeline tablets (0, 7.5, 15, or 30 mg, s.l.) after 1 h. Inactive doses of both sublingual and oral formulations were used as placebo. They observed that lobeline could improve working memory in a modest capacity (without significant improvement in their attention) in nine volunteers with ADHD.

Li et al. investigated the effect of s.c administrations of lobeline hydrochloride (1 or 3 mg/kg) on male C57BL/6J mice against methyl-4-phenyl-1,2,3,6-tetra-hydropyridine (MPTP, 30 mg/kg)-induced cell death *in vivo* using L-dopa (80 mg/kg) and the potent and selective dopamine reuptake inhibitor, GBR12935 (10 mg/kg) as positive controls (Li et al., 2014). The s.c. injections of lobeline, GBR12935, or L-dopa were done for 11 days 30 min prior to MPTP administration. They observed that MPTP induced locomotive effects detected in behavioral tests were significantly reduced by lobeline as compared to L-dopa and GBR12935. Further, they observed that the loss of neurotoxin-induced immunoreactivity was significantly reduced in the substantia nigra and striatum regions of the brain. The overall results indicated that lobeline may protect dopaminergic neurons and alleviate the symptoms of Parkinson’s disease. Saline was used as the negative control.

Tamboli et al. investigated lobeline isolated from *Lobelia* nicotianaefolia in chemoconvulsant-induced seizures using diazepam as a reference standard (Tamboli et al., 2012). The anticonvulsant activity of the isolated lobeline was investigated at 5, 10, 20 and 30 mg/kg, intraperitoneal (i.p.) doses in Pentylenetetrazol (PTZ) and strychnine induced seizures in Swiss albino male mice. They found that isolated lobeline induces potent anticonvulsant activity against PTZ induced seizures by enhancing the brain gamma amino butyric acid (GABA) level at 20 mg/kg i.p. within a duration of 45 min after their administration. Saline was used as the negative control.

Mechanistically, lobeline was reported binding to the α4β2 nAChR with nanomolar affinity (Brioni et al., 1997) but with less potent affinity against muscarinic and acetylcholinesterase receptors (Terry et al., 1998). The high-affinity partial agonist property of lobeline against α4β2 nAChR was later identified as the mechanism for smoking cessation (Wu et al., 2006; Billen et al., 2012). Recently, Anitha et al. shed additional light on how smoking cessation by lobeline occurs through trapping in α4β2 receptors-containing acidic vesicles and how intracellular pH homeostasis plays a crucial role in it (Govind et al., 2017). Lobeline was also demonstrated to block the blood-brain barrier (BBB) basic amine transporter (Allen et al., 2003), and act as VMAT2 ligand (Zheng et al., 2005; Zheng et al., 2006). In another study, (−)-lobeline hydrochloride monohydrate (10 or 20 mg/kg, i.p.) was reported to reduce the DNA damage and oxidative stress induced by seizures in a pilocarpine-induced seizure model on 69 adult male CF-1 mice (da Costa E Silva et al., 2018). The administrations of pilocarpine (300 mg/kg) were performed after 30 min treatment of lobeline, diazepam (positive control) or saline (negative control).

Overall, to date, more than 610 scientifically peer-reviewed research articles carrying the pharmacological investigation of the pyridine alkaloid lobeline and its structural analogs were reported underlying its medicinal importance. However, the gastrointestinal side effects (due to nicotinic effects), the lethal toxicity due to its narrow therapeutic index, and other adverse side effects such as choking and cough limits the clinical utility of lobeline (Butler et al., 2001; Raj et al., 2005; Stead and Hughes, 2012; Heinzerling, 2013).

### Beyond Lobeline: Other Bioactive Alkaloids

In the early 1950s, the German phytochemist Steinegger and team reported several key discoveries on the identification and pharmacological studies of the newer alkaloids from different *Lobelia* species. First, they reported the identification of a crystallized alkaloid named lurenine isolated from *Lobelia urens* (Steinegger and Grutter, 1950)*.* Subsequently, they also identified two new alkaloids lophilin and lophilacrin in the alkaloid fraction of *Lobelia siphilitica* (Steinegger and Egger, 1952). In the 1950s, other studies began to reveal the distinctive pharmacological properties of the alkaloid constituents identified in different *Lobelia* species (Lendle and Richter, 1950), which has further raised tremendous interest in phytochemical research. Lobinaline 2, the only alkaloid found in the *Lobelia cardinalis* L. and the first binitrogenous alkaloid extracted from any *Lobelia* species was reported having a unique nAChR binding activity (Klosa, 1953; Kaczmarek and Steinegger, 1958; Brown et al., 2016). In addition to their equipotent α4β2-and α7-nAChRs binding profile, the purified lobinaline obtained from CHCl_3_ fraction of air-dried aerial portions of *L. cardinalis* also exhibited potent 2,2-Diphenyl-1-picrylhydrazyl (DPPH) free radical scavenging properties, inhibited *in vitro* [(3)H]-dopamine uptake in adult male Sprague-Dawley rats striatal synaptosomes, and potent dopamine transporter inhibition (Brown et al., 2016). Reportedly, lobinaline (stock solution: 1 mg/ml) scavenge DPPH free radicals with an EC_50_ of 17.98 μM when examined in DPPH (stock solution: 600 μM) free radical scavenging assay. In contrast, lobeline (stock solution: 1 mg/ml) demonstrated a weak DPPH free radical scavenging property (EC_50_ = 228.8 μM). *In vivo* electrochemical studies indicated that lobinaline in a short acting and likely competitive-capacity inhibits dopamine transporter function in the dorsal striatum of urethane-anesthetized rats. Interestingly, lobinaline (1 mM) was noticed significantly increasing ^45^Ca^2+^ entry in SH-SY5Y cells.

In 1954, major alkaloids named lobelanidine 3, and lelobanidine 4 were also isolated from *Lobelia nicotianaefolia* (Gedeon and Gedeon, 1954). Lobelanidine 3 was subsequently identified in *Lobelia chinensis* exhibiting anticancer and anti-emetic properties (Chen et al., 2014). In 2001, two new pyrrolidine alkaloids, radicamines A 5 and radicamines B 6 isolated from the hot water extract of *Lobelia chinensis* Lour. (Shibano et al., 2001). The basic extract solution (25 µL) was were identified exhibiting potent alpha-glucosidase (100 µL) inhibition (Shibano et al., 2001). Radicamines A, radicamines B and the positive control (2*R*,3*R*,4*R*,5*R*) 2,5-dihydroxymethyl-3,4-dihydroxy-pyrrolidine (DMDP) exhibited IC_50_ of 6.7 × 10^–6^, 9.3 × 10^–6^, and 4.9 × 10^–6^ M, respectively.

In 2015, Paz et al. reported a new alkaloid, pentylsedinine 7, isolated from the fresh aerial parts of *Lobelia tupa* (Paz et al., 2015). When evaluated against the α3β2/α3β4 nAChR and α7 nAChR receptors expressed in the human neuroblastoma cell line SH-SY5Y, like lobeline, (-)-pentylsedinine acted as a non-selective partial agonist at homomeric α7 nAChR (EC_5_0 0.4 ± 0.08 µM) and heteromultimeric α3β2/α3β4 (EC_50_ 0.33 ± 0.07 µM) nAChR. Interestingly, the co-addition of lobeline and (-)-pentylsedinine desensitized α7 nAChR to the response from the selective α7 nAChR agonist choline. Apparent IC_50_ of lobeline and (-)-pentylsedinine were 1.6 ± 0.4 µM and 0.37 ± 0.08 µM, respectively.

Lobelane 8, a defunctionalized analog of lobeline exhibited 10–15 fold more potent effects than lobeline in inhibition of the vesicular dopamine (DA) uptake by VMAT2, and inhibited (+)-methamphetamine-evoked DA release (Vartak et al., 2009; Nickell et al., 2010). It was also reported to possess a fivefold binding affinity than lobeline for the dihydrotetrabenazine binding site on VMAT2 (Miller et al., 2004; Zheng et al., 2005). In a study aimed at synthesizing lobelane analogs as therapeutic agents, replacement of the phenyl groups of lobelane with quinolyl groups resulted in a water-soluble analog called quinlobelane nine that possessed stronger VMAT2 inhibition (Vartak et al., 2010).

The phytochemical research conducted over the years suggested that alkaloids are the predominant secondary metabolites (∼46%) present in most of the *Lobelia* species known. These alkaloids ubiquitously contain *N*-methylpiperidine ring or piperidine ring along with one or two substituents at the C_2_ and/or C_6_ position of the ring (Felpin and Lebreton, 2004; Folquitto et al., 2019).

**FIGURE 2:**
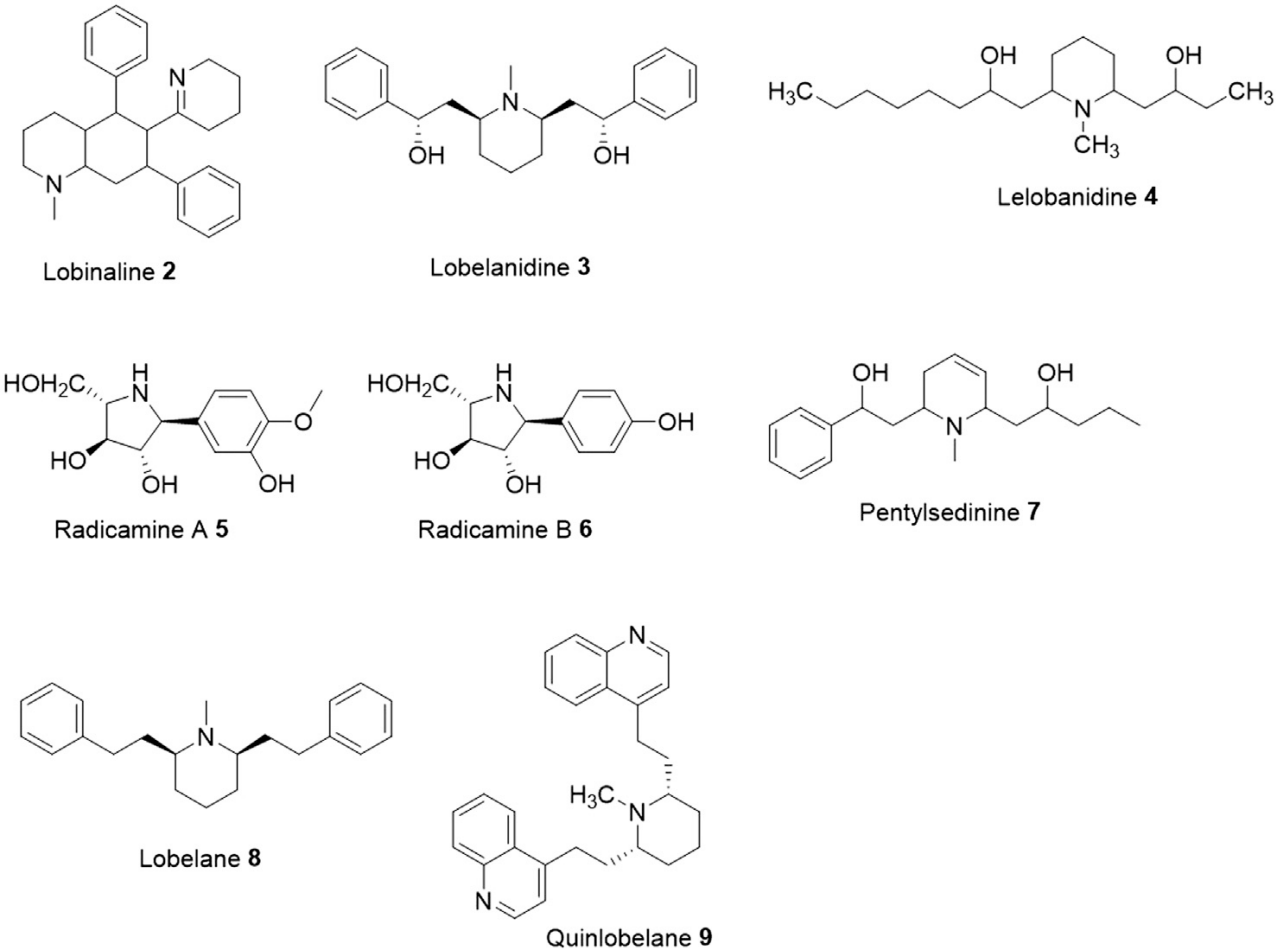
Chemical structures of the bioactive alkaloids derived from *Lobelia* species.

One of the major disadvantages of the alkaloids derived from the *Lobelia* species has been their extreme resemblance in the chemical structures to each other, essentially sharing similar pharmacological, physicochemical, and toxicological profiles which also include cardiotoxicity (Figure 2). The alkaloids in most of the species of *Lobelia* (with lobeline being the most cited in the literature) are broadly known for presenting medicinal properties against drug abuse, neurological disorder, and respiratory stimulant. The limited medicinal properties of these alkaloids can be attributed to their binding properties at the nAChR receptors. In this present manuscript, we have only considered the publications where the alkaloid was biologically tested. Unfortunately, the bioactivity characterization of a large majority of those alkaloids was not yet reported and thus excluded. Moreover, to date, more than 80 alkaloids are known to be isolated from different species of *Lobelia.* The readers are recommended to the following review for the updates on other isolated alkaloids (Folquitto et al., 2019).

### Beyond Alkaloids: The Bioactive Components of Other Classes

Over the years, the isolation and characterization of secondary metabolites derived from *Lobelia* species have garnered significant attention. It is now an established fact that most species of the genus *Lobelia* contain different classes of secondary metabolites including but not limited to flavonoids, terpenoids, fatty acids, polyacetylenes, coumarins, neolignans, polysaccharides, and amides in varying concentrations. In recent years, the identifications of these newer compounds also presented the opportunities to address the limited bioactivity of alkaloids mentioned earlier. Due to this fact, growing numbers of literature are being published. In this section, we will present a current account of these newer novel bioactive natural products derived from *Lobelia* species. A special focus has been given to accounting for the molecular mechanism of these newer secondary metabolites.

Since mid-to end- 20th-century investigations into the pharmacology of components derived from *Lobelia* species were centered onto the alkaloids. In 1961, Sasaki et al. reported the isolation of a new glucofructan “sessilifolan” from *Lobelia sessilifolia* Lamb. I. (Sasaki et al., 1961; Sasaki and Mikami, 1962). It was perhaps the first report identifying other secondary metabolites than alkaloids in *Lobelia*. In 1986, Santosa et al. reported the *in vitro* as well as *in vivo* activity of the hot water extract of *Lobelia chinensis* against Ehrlich ascites tumor and Sarcoma 180 cells (Santosa et al., 1986). However, in subsequent decades, not much progress has been made on characterizing the other classes of secondary metabolites.

Subarnas et al. identified a novel antidepressant bioactive component, β-amyrin palmitate 10 in an extract of the leaves of *Lobelia inflata* (Subarnas et al., 1992). Further studies indicated that the antidepressant activity of β-amyrin palmitate 10 was related to the activation of the noradrenergic system by releasing [3H]norepinephrine from newly synthesized pools, with mianserin-like action (Subarnas et al., 1993b; Subarnas et al., 1993c). In a forced swimming test, β-amyrin palmitate at 10 mg/kg reduced the tetrabenazine-induced (100 and 200 mg/kg) increase in the duration of the immobility of mice whereas it has shown no effect when treated with alpha-methyl-para-tyrosine (500 mg/kg). Similarly, at 5 and 10 mg/kg, it has decreased the duration of immobility in mice treated with 6-hydroxy-dopamine (50 µg/mouse) and desipramine (15 mg/kg) without showing any effect to the treatment of mice with nomifensine plus 6-hydroxydopamine (Subarnas et al., 1993b). Physicochemically, beta-amyrin palmitate is a water-insoluble, extremely weak basic compound belonging to the triterpenoids compound class which contains six isoprene units. Other studies reported β-amyrin palmitate exhibiting different pharmacological properties such as induction of hypoactivity by inhibiting alpha 1-adrenoceptors (Subarnas et al., 1993a), anti-diabetic and anti-hyperglycemic activity by blocking the entry of glucose from the intestine (Nair et al., 2014), and antibacterial against Gram-positive and negative bacteria and antifungal activities (Abed et al., 2016).

Recently, Wang et al. reported the identification of four novel flavonoid glycosides, lobelitin A-D 11–14 exhibiting agonistic activities against G protein-coupled receptor 35 (GPR35) (Wang et al., 2019). The compounds were isolated from *Lobelia chinensis*.

**FIGURE 3:**
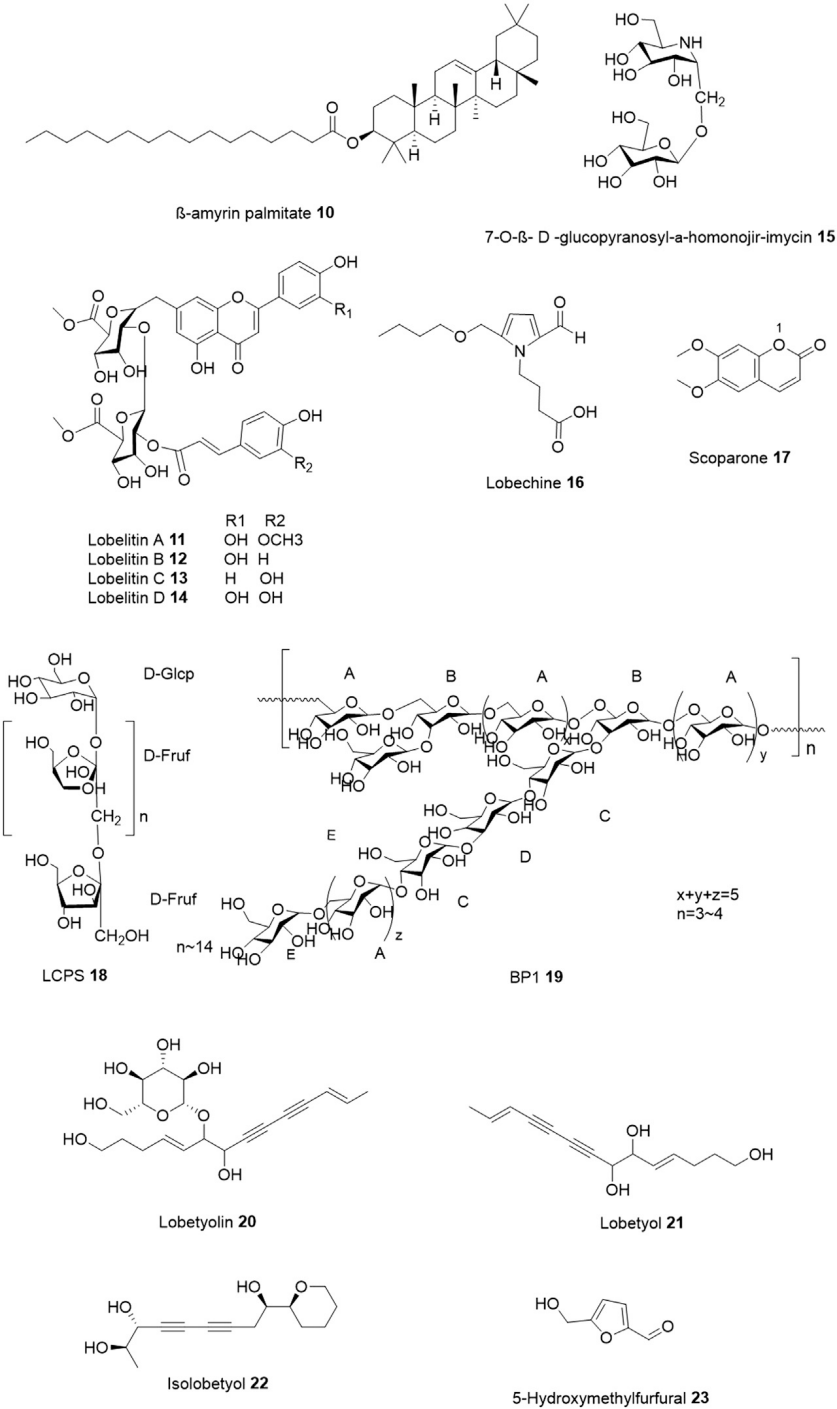
Chemical structures of non-alkaloids novel bioactive compounds isolated from *Lobelia* species.

The authors noticed that lobelitin A-D triggers dose-dependent positive signals (monitored for 1 h) in dynamic mass redistribution (DMR) agonist assay in varying concentration. For example, at 200 pm, lobelitin A, B, and D were recorded exhibiting the largest DMR signal whereas at about 100 pm, lobelitin C exhibited a weaker signal at the maximum concentration of 250 µM. Based on these results, the authors suggested lobelitin A, B and D as the full agonist and lobelitin C as a partial agonist of GPR35. The structures and the apparent EC_50_ and IC_50_ values of lobelitin A-D are shown in Figure 3 and Table 1. GPR35 has been implicated in several diseased conditions including hypertension, coronary artery disease, and cancer, and is an area of active research (Divorty et al., 2015; Wei et al., 2017; Quon et al., 2020).

**TABLE 1:** Pharmacological characteristics of active flavonoid glycosides derived from *Lobelia chinensis* in HT-29 cells (Wang et al., 2019).

Compounds	EC_50_ (µM)	IC_50_ ^a^ (µM)	IC_50_ ^b^ (µM)
Lobelitin A	49.92 ± 12.61	0.38 ± 0.08	47.63 ± 6.31
Lobelitin B	18.77 ± 2.02	0.27 ± 0.03	27.89 ± 2.33
Lobelitin C	Weak	0.39 ± 0.21	72.74 ± 22.96
Lobelitin D	67.65 ± 37.99	0.48 ± 0.13	64.70 ± 17.19

^a^ IC_50_ of ML145 to block the DMR of compounds.

^b^ IC_50_ to desensitize the DMR of 1 µM zaprinast in HT-29 cells.

The glucoside 2,6-Dideoxy-7-O-(b- D -glucopyranosyl) 2,6-imino- D -glycero- L -gulo-heptitol (7-O-β-D-glucopyranosyl-α-homonojir-imycin, 15) isolated from the methanol extract of the whole plant of *Lobelia sessilifolia* exhibited potent inhibitory activity against various α-glucosidases (Ikeda et al., 2000). Specifically, 7-*O*-β- D -glucopyranosyl-α-homonojir-imycin exhibited an IC_50_ of 0.25and 0.27 µM against rice and rat intestinal isomaltase α-glucosidase, respectively. Additionally, it has also shown the inhibition of porcine kidney trehalase (IC_50_ = 0.013 µM) and anti-diabetic property (Ikeda et al., 2000).

Kuo et al. reported the antivirus and anti-inflammatory properties of a novel compound lobechine 16 alongwith scoparone (6,7-dimethoxycoumarin) 17 isolated from the methanol extracts of *Lobelia chinensis*. Lobechine 16 inhibited elastase release with IC_50_ of 25.01 ± 6.95 μM whereas scoparone 17 inhibited superoxide anion generation with IC_50_ of 6.14 ± 1.97 μM (Kuo et al., 2011).

A neutral polysaccharide (LCPS) 18 was isolated from *Lobelia chinensis* lour. The monosaccharide composition of LCPS was observed as fructan composed of 2,1-linked-β-D-fructofuranosyl residue and α-D-glu-copyranosyl residue with α-D-Glcp-(1→[1)-β-D-Fruf-(2]_15_ glycosidic linkage. LCPS was identified as an inulin-type fructan with 2.6 kDa molecular weight with an effective therapeutic option in the treatment of obesity. Oral administration of this inulin alleviated obesity and reduced body weight in high-fat diet-induced mice (Zhang et al., 2020). Specifically, the anti-obesity effects of LCPS evaluated *in vivo* in male Kunming mice treated with normal chow diet (D12450 J), high fat diet (D12492) and high fat diet plus LCPS (300 mg/kg/day) for eight weeks showed 67% lower body weight gain as compared to high fat diet treatment. High fat diet plus glibenclamide (5 mg/kg/day) was used as a positive control which has shown 77% effect.

**FIGURE 4:**
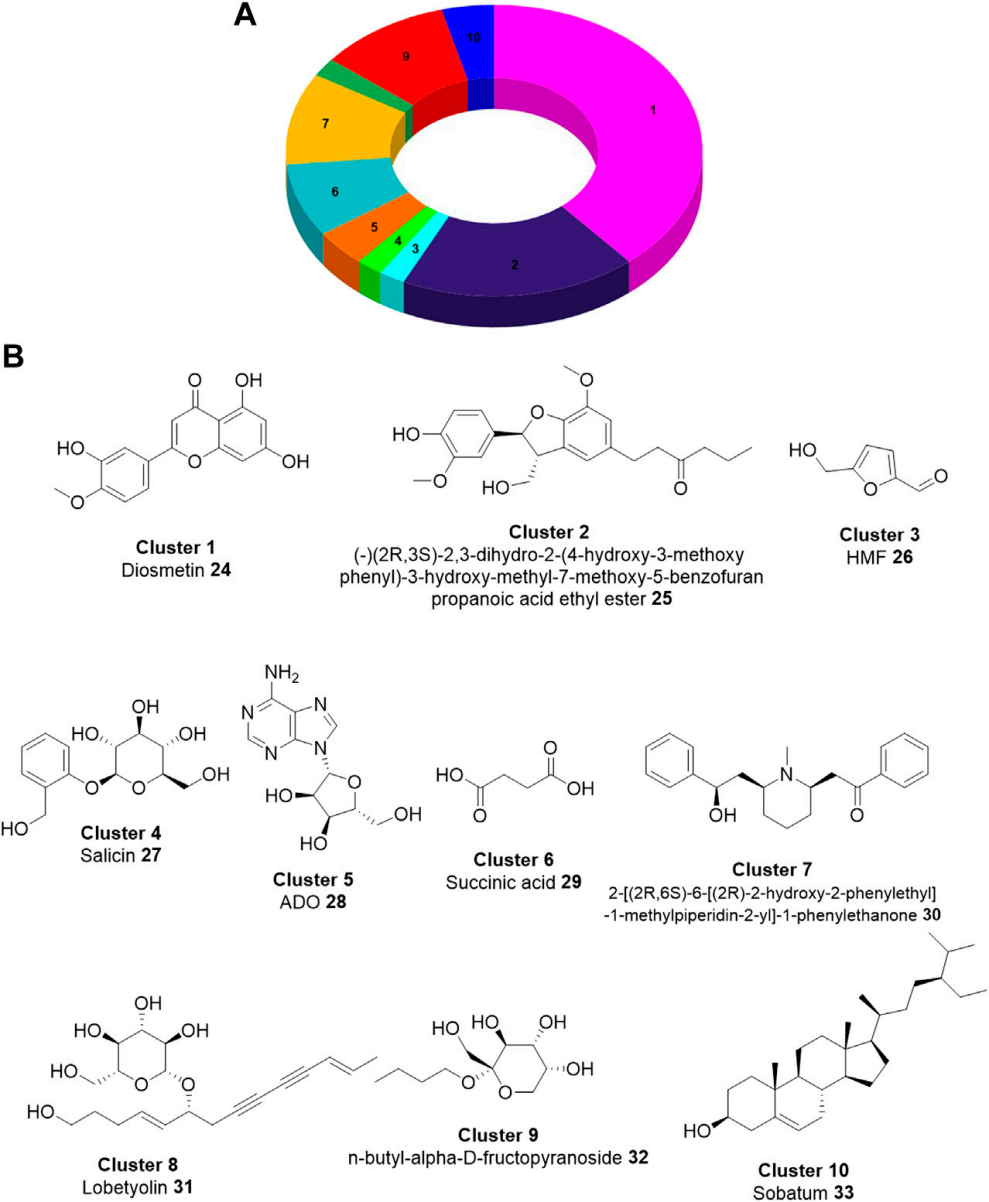
Chemical diversity of the components derived from 17 *Lobelia* species. **(A)**. The distribution of the chemical compounds in the top 10 clusters based on ECFP4 fingerprint. **(B)**. Chemical structure of the representative compound in individual clusters (Zheng et al., 2020).

Li et al. isolated another neutral α-glucan, named BP1 19 (Mol. weight: 9.45 kDa), from *Lobelia chinensis* by hot-water extraction and studied its immunomodulating activities (Li et al., 2016). The authors proposed that α-glucan BP1 activates Toll-like receptor 4 (TLR4) and exerts immunomodulating effects such as enhancement of the cell proliferation, nitric oxide production, cytokine secretion and phagocytosis in a dose-dependent manner. Additionally, the glucan also induced cytokine and nitric oxide production mediated by toll-like receptor 4 (TLR4) in RAW 264.7 cells. The backbone of BP1 consisted of alternating units of _6_α- D -Glcp^1^ -- _6,3_α- D -Glcp^1^-- (_6_α- D-Glcp^1^)x--(_6,3_α- D -Glcp^1^)y. The side chains were α-D-Glcp^1^ --(_6_α-D-Glcp^1^)z--_4_α-D-Glcp^1^--_3_α-D-Glcp^1^--_4_α-D-Glcp^1^ and α- D-Glcp^1^ terminally attached to backbone O-3 of _6,3_α- D -Glcp^1^ (as shown in Figure 4). Specifically, BP1 demonstrated a dose-dependent induction of proliferation of RAW 264.7 cells at the concentrations of 12.5, 25, 50, and 100 µg/ml when administered together with polyB in an MTT assay and tested for 24 h.

Ishimaru et al., isolated two new polyacetylene compounds lobetyolin 20 and lobetyol 21 from the hairy roots of *Lobelia inflata* (Ishimaru et al., 1991)*.* Recently, He et al. studied the effect of lobetyolin on colorectal cancer (He et al., 2020). They found that lobetyolin induces colon cancer cells by inhibiting the ASCT2-mediated glutamine metabolism. Briefly, they have incubated lobetyolin in HCT‐116 cells for 3, 6, 12, 18 and 24 h at varying concentrations of 10, 20 and 40 μmol/L in an MTT assay and observed that lobetyolin induces largest inhibitory effect on survival rate at 24 h. On the other hand, the authors did not observe any significant influence in the cell viability in NCM460 cells at 10, 20 and 40 μmol/L. Additionally, they observed that lobetyolin at 10, 20, and 40 μmol/L prominently suppresses the expression of ASCT2 protein and reduces the mRNA levels. In addition, lobetyolin at 40 μmol/L also inhibited the expression of ASCT2 in HCT-116 cells. Further, lobetyolin (10, 20, and 40 mg/kg) inhibited the tumor volume in the nude (nu/nu) mice transplanted with HCT‐116 cells. In another study, Shen et al. reported that lobetyol induces anti-carcinoma effect in MKN45 cells and activates MAPK pathways associated with G_1_/S cell cycle arrest in a time- and dose-dependent manner (Shen et al., 2016). Specifically, the anti-proliferative effect of lobetyol in MKN45 cells was evaluated in an MTT assay at a varying concentration and time ranging from 50 µg/ml, 75 µg/ml, to 110 µg/ml and 12 h, 24 h, to 48 h, respectively. Lobetyol demonstrated an IC_50_ of 71.47 ± 4.29 µg/ml at 48 h. Further, in MKN45 cells, an increasing dose of lobetyol ranging from 0, 50, and 75 µg/ml, to 110 µg/ml led to an increase in apoptotic population by 5.5%, 13.74%, 27.32%–31.57%, respectively.

Two other polyacetylene isolobetyol 22 and lobetyol 21 isolated from *Lobelia chinensis* exhibited moderate cytotoxic activities against MSTO-211H and NCI-H292 cell lines (treated for 48 h). Isolobetyol exhibited the IC_50_ values of 12.36 and 9.31 μM against the two cell lines; while the lobetyol showed the IC_50_ values of 11.76 and 9.64 μM, respectively (Yang et al., 2014). In contrast, positive control (cisplatin) showed an IC_50_ of 4.91 and 7.89 μM against MSTO-211H and NCI-H292 cell lines, respectively. However, the authors did not mention at which concentrations the compounds were tested.

In another report, Joshi et al. analyzed the essential oil composition of the aerial parts of *Lobelia pyramidalis* Wall and evaluated their antimicrobial activity (Joshi et al., 2011). Serial doubling dilutions of the essential oil were prepared from 0.10 to 200 mg/ml and incubated in Mueller Hinton broth for the bacterial strains at 37°C for 24 h. The minimal inhibitory concentration and inhibition zone of 3.12 mg/ml and 18 mm, respectively was determined against the bacterial strain *S. aureus*. The authors suggested natural terpenoid perilla ketone could be the responsible chemical constituent. However, no further attempt was made to isolate and characterize the compound.

In a recent study, Ma and colleagues identified 208 metabolites of *Lobelia chinensis* by gas chromatography-mass spectrometer (GC/MS) and conducted a network pharmacology-based investigation of their anti-diabetic mechanism (Ge et al., 2020). Their study revealed that 5-hydroxymethylfurfural 23 acts on GSK3B, TNF, and MAPK1 and affects the insulin resistance signaling pathway as the major mechanism of action. Also, acacetin might act on INSR, DPP4, and GSK3B. No biological evaluation was performed to validate the network pharmacology approach’s presented findings, which remains one major drawback of this work.

We have also recently investigated the neuroprotection mechanism of the pharmacokinetically favorable, experimentally validated active compounds derived from the 17 *Lobelia* species (Table 2) based on network pharmacology and molecular modeling (Zheng et al., 2020). In this work, we also studied the chemical diversity of the validated natural compounds identified in the genus of the *Lobelia* species including the major components of *Lobelia chinensis* studied by Ma and Colleagues (Ge et al., 2020). Fascinatingly, the aglycone part of the flavonoid such as diosmetin and 18 other aglycones constituted the densest cluster indicating their profound presence in the *Lobelia* species. The representative chemical structure of the top 10 clusters is shown in Figure 4B. We have also not performed the biological evaluations. However, in this review, we intended to summarize the experimentally determined chemical constituents of *Lobelia* and draw a comparison in their chemical structures (as shown in Figure 4).

**TABLE 2:** *Lobelia* species used in our study to investigate the neuroprotection mechanism.

NPASS IDs	Herb name	Family	Super kingdom	# Of natural product	References
NPO10220	*Lobelia davidii*	Campanulaceae	Eukaryota	3	Zhang et al. (1990)
NPO11547	*Lobelia cardinalis*	Campanulaceae	Eukaryota	4	Kaczmarek and Steinegger (1958), Kelley et al. (2019)
NPO11565	*Lobelia salicifolia*	Campanulaceae	Eukaryota	7	Steinegger and Ochsner (1956)
NPO14650	*Lobelia chinensis* ^a^	Campanulaceae	Eukaryota	71	Shibano et al. (2001), Zhou et al. (2008), Kuo et al. (2011), Yang et al. (2014), Li et al. (2016), Wang et al. (2019), Kuo et al. (2011), Ge et al. (2020)
NPO14996	*Lobelia syphilitica*	Campanulaceae	Eukaryota	20	Kesting et al. (2009)
NPO15709	*Lobelia langeana*	Campanulaceae	Eukaryota	20	
NPO16059	*Lobelia polyphylla*	Campanulaceae	Eukaryota	2	Weinges et al. (1972)
NPO17414	*Lobelia portoricensis*	Campanulaceae	Eukaryota	1	Meléndez et al. (1967)
NPO18202	*Lobelia radicans* ^a^	Campanulaceae	Eukaryota	5	
NPO238	*Lobelia st*	Campanulaceae	Eukaryota	16	
NPO24373	*Lobelia inflata*	Campanulaceae	Eukaryota	20	Ishimaru et al. (1991), Bálványos et al. (2004), Kursinszki et al. (2008)
NPO26111	*Lobelia camporum*	Campanulaceae	Eukaryota	1	
NPO293	*Lobelia nicotianifolia*	Campanulaceae	Eukaryota	1	
NPO29542	*Lobeliae chinensis herba* ^a^	Campanulaceae	Eukaryota	41	
NPO28802	*Lobelia nummularia* lam	Campanulaceae	Eukaryota	3	Ishimaru et al. (1991), Ho et al. (1995)
NPO5012	*Lobelia hassleri*	Campanulaceae	Eukaryota	4	
NPO5051	*Lobelia urens*	Campanulaceae	Eukaryota	3	
NPO5103	*Lobelia berlandieri*	Campanulaceae	Eukaryota	4	Williams et al. (1987)
NPO615	*Lobelia exaltata*	Campanulaceae	Eukaryota	1	
NPO8894	*Lobelia fistulosa*	Campanulaceae	Eukaryota	2	

^a^ are the same species with two different entries in NPASS database. (Zeng et al., 2018)

### Data Sources and Study Selection

The literature search was performed using PubMed and Scifinder over the period from the beginning of the database until October 2020. Initially, all the literature associated with the search term “*Lobelia*” were collected. In addition, the databases were also directly searched using the botanical name (e.g., “*Lobelia chinensis*”). In addition, as a third search strategy, the databases of the major research journals including Science Direct, Frontiers, Wiley, American Chemical Society, MDPI, Springer-Nature, and Taylor and Francis were also searched manually. Articles that have reported only isolation and identification of chemical constituents without biological activities were excluded. No unpublished literature data or conference proceedings were included. Except for a recent systematic review by Folquitto et al. (Folquitto et al., 2019), other articles that described reviews or systematic reviews were excluded. The systematic review by Folquitto et al. was used as an additional strategy to validate the references for the phytochemical details.

## Conclusion

Undoubtedly, *Lobelia* is an important genus with profound medicinal value. A lot of work has already been done in understanding the pharmacological role of the alkaloids derived from different *Lobelia* species. Yet, growing investigations on the newer chemical classes indicate that there is certainly an untapped opportunity to obtain bioactive compounds with diverse chemical and pharmacological properties. In this work, we tried to provide the readers the most up-to-date account of the progress made so far, especially toward understanding the molecular mechanism and pharmacological characterizations of the novel bioactive components. Considering the progress being made in the last few years, we believe that this is a timely review to provide the scientific basis for future research aimed at studying the therapeutic potential of the newer chemical compounds derived from the *Lobelia* species.
